# Delayed cerebral radiation necrosis following treatment for a plasmacytoma of the skull

**DOI:** 10.4103/2152-7806.71984

**Published:** 2010-10-25

**Authors:** Lola B. Chambless, Federica B. Angel, Ty W. Abel, Fen Xia, Kyle D. Weaver

**Affiliations:** Department of Neurological Surgery, Vanderbilt University Medical Center, Nashville, TN, USA; 1 Department of Internal Medicine, Vanderbilt University Medical Center, Nashville, TN, USA; 2 Department of Pathology, Division of Neuropathology, Vanderbilt University Medical Center, Nashville, TN, USA; 3 Department of Radiation Oncology, Vanderbilt University Medical Center, Nashville, TN, USA

**Keywords:** Brain, multiple myeloma, plasmacytoma, radiation necrosis, radiotherapy

## Abstract

**Background:**

Cerebral radiation necrosis is a relatively common complication of radiation therapy for intracranial malignancies which can also rarely be encountered after radiation of extracranial lesions of the head and neck. We present the first reported case of cerebral radiation necrosis in a patient who underwent radiation therapy for a plasmacytoma of the skull.

**Case Description:**

A 68-year-old male with multiple myeloma presented with an enhancing right frontal mass, 8 years after receiving radiation therapy for a plasmacytoma of the left frontal skull. The patient underwent a diagnostic and therapeutic craniotomy for a presumed neoplastic lesion. The pathologic diagnosis made in this case was delayed radiation necrosis. The patient was followed for over a year during which this process continued to evolve before the ultimate resolution of his clinical symptoms and radiographic abnormality.

**Conclusion:**

This case highlights the importance of considering radiation necrosis in the differential diagnosis of any patient with an intracranial mass and a history of radiation for an extracranial head and neck malignancy, regardless of timing and laterality. This case also provides unique insights into the ongoing debate regarding the role of the aberrant immune response in the pathogenesis of delayed cerebral radiation necrosis.

## INTRODUCTION

Cerebral radiation necrosis is a well-recognized complication of radiation therapy for intracranial neoplasms. Actuarial data are limited, but this complication is reported to occur in 3–6% of patients undergoing standard therapy for gliomas in most studies and was reported to be as high as 24% in studies examining aggressive chemoradiation protocols for intrinsic intracranial lesions.[1] The incidence of cerebral radiation necrosis peaks 1–3 years after treatment.[2] Delayed cerebral radiation necrosis, generally defined as radiation necrosis which develops more than 3 years after completion of treatment, is far more unusual, but has been reported to occur as many as 47 years after radiation therapy for an intracranial lesion.[3]

The occurrence of cerebral radiation necrosis following radiation for extracranial malignancies is uncommon but has been reported. The majority of these cases have been diagnosed within 2 years of treatment. This complication has been described in patients treated with radiation therapy for cutaneous squamous cell carcinoma of the scalp as well as carcinomas of the lacrimal gland, sinuses, and orbit.[4–7] There is also a well-described phenomenon of cerebral radiation necrosis occurring after treatment for nasopharyngeal carcinoma.[8] Delayed radiation necrosis presenting as an intracranial mass identified 19 years after treatment has also been described in one patient with a history of radiation therapy for a basal cell epithelioma of the scalp.[9]

To our knowledge, the case presented here is the first report of cerebral radiation necrosis occurring, either in a routine or delayed fashion, following radiation for a plasmacytoma of the skull. This case represents a unique presentation of a treatment complication in a patient with multiple myeloma and highlights a challenging neurosurgical diagnosis.

## CASE REPORT

**Figure 1:**
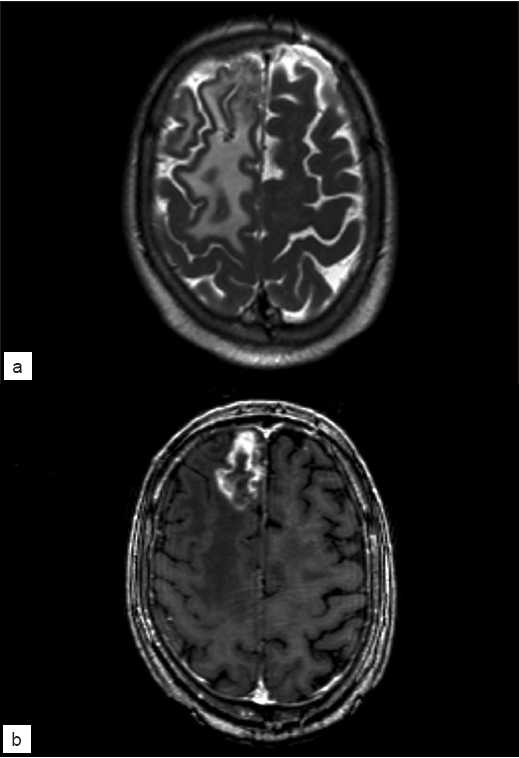
T2 (a) and T1 post-contrast (b) MRI obtained at the time of presentation, demonstrating edema throughout the right fronto-parietal region with an enhancing, necrotic lesion within the anterior right frontal lobe. These imaging features are typical of radiation necrosis

A 68-year-old, right-handed male presented to his primary care physician with several months of gradually progressive cognitive changes, gait disturbance, and headache. Magnetic resonance imaging (MRI) of the brain revealed a heterogenously enhancing lesion in the anteromesial right frontal lobe which was associated with significant edema [Figure 1a and b]. The patient was immediately referred for neurosurgical evaluation.

The patient’s past medical history was considered closely. The patient was diagnosed with a plasmacytoma of the left frontal skull 8 years prior to this presentation and was initially treated with radiation therapy to this isolated lesion. This treatment took place at an outside institution and involved conventional external beam radiation using a 20 MeV electron beam. The initial radiation treatment was delivered with a 10 cm × 10 cm cone, at 100 SSD, with the beam positioned perpendicular to the left frontal scalp and with a 0.5 cm wet bolus. The patient received 2.5 Gy per fraction for 7 fractions, followed by decreased dose of 2.0 Gy per fraction for additional 11 fractions. The total cumulative dose to this treatment field was 39.5 Gy. The treatment field was then coned down to 6 cm × 6 cm, with three more fractions of radiation treatment at 2.0 Gy per fraction. The total accumulated dose to the boost field was thus 45.5 Gy.

Shortly after the conclusion of radiation therapy, the patient developed additional sites of systemic disease and underwent an autologous stem cell transplant for multiple myeloma. He remained disease-free since that time and was treated with chronic immunosuppressive agents to prevent rejection.

Upon neurosurgical evaluation, a radiographic differential diagnosis was formulated and included high-grade glioma, metastasis, abscess, radiation-induced malignancy, and radiation necrosis. Radiation necrosis was considered a relatively unlikely diagnosis due to the timing of the presentation. Additionally, the reported total radiation dose to the brain parenchyma was low and was divided into numerous small fractions and the plasmacytoma targeted with radiation was contralateral to the new intrinsic lesion. However, the detailed radiation plan from the original treating facility was destroyed, and without the ability to review this document, treatment error was considered possible. The use of additional imaging modalities such as MR spectroscopy or FDG-Positron Emission Topography (PET) was considered, but these results were considered unlikely to change the treatment recommendations. After a discussion with the patient and his family, the decision was made to proceed with a craniotomy for pathologic diagnosis and relief of mass effect without further preoperative workup.

### Operation

Shortly after referral, the patient underwent a right frontal craniotomy for resection of the mass lesion. Frameless stereotaxy was used to localize the lesion which was resected with conventional microsurgical techniques. The lesion was noted to be firm and white and extended medially to the pial border of the interhemispheric fissure. A large central portion was delivered in its entirety and sent to neuropathology for frozen and permanent section. The frozen section was notable for hyalinized blood vessels, calcification, and an absence of gross cellular atypia. With a pathologic impression favoring radiation necrosis, the craniotomy was completed with a goal of internal debulking of the lesion for relief of mass effect.

### Pathology

**Figure 2:**
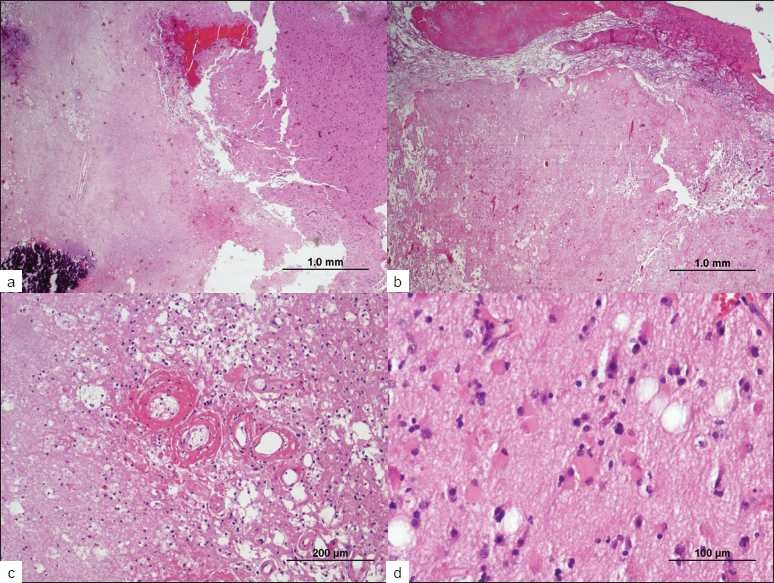
Permanent pathologic specimen demonstrating coagulative necrosis at low power (a,b) with coarse calcium deposits (a) and leptomeningeal fibrosis (b) At high power, hyalinized blood vessels are apparent within regions of necrosis (c) and areas of gliotic brain tissue are apparent with modest hypercellularity and cellular atypia (d) The features are consistent with a diagnosis of cerebral radiation necrosis

The entire specimen, consisting of large fragments of tissue measuring approximately 4 cm in aggregate, was submitted for microscopic examination. This showed extensive bland, granular, coagulative necrosis, with smaller areas of viable, reactive brain tissue [Figure 2a–d]. Areas of necrosis contained hyalinized blood vessels [Figure 2c] and coarse calcium deposits [Figure 2a]. Few macrophages were present. Viable brain tissue was gliotic but only modestly hypercellular, with moderately atypical cells [Figure 2d] and benign, perivascular chronic inflammation. Focal capillary proliferation resembling a telangiectasia was present. There was no evidence of malignancy or infection. A diagnosis of radiation-induced necrosis was made.

### Postoperative course

Postoperatively, the patient returned to his neurologic baseline. A postoperative MRI demonstrated near-total resection of the lesion with some residual nodular enhancement. After a fall from standing, the patient developed a wound complication requiring exploration on postoperative day 2, in which a subgaleal hematoma was identified and evacuated. He subsequently recovered well and was discharged to a rehabilitation facility several days later.

**Figure 3:**
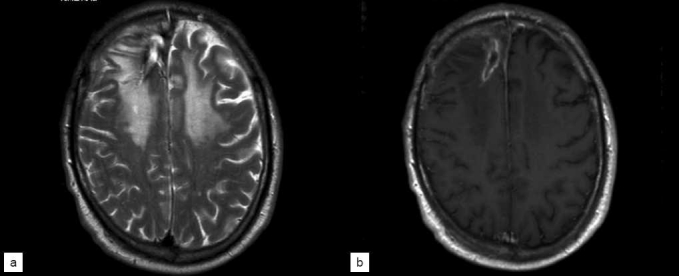
T2 (a) and T1 post-contrast (b) MRI obtained 6 months after craniotomy, demonstrating continued evolution of the right frontal necrotic lesion as well as bifrontal edema consistent with radiation effect

**Figure 4:**
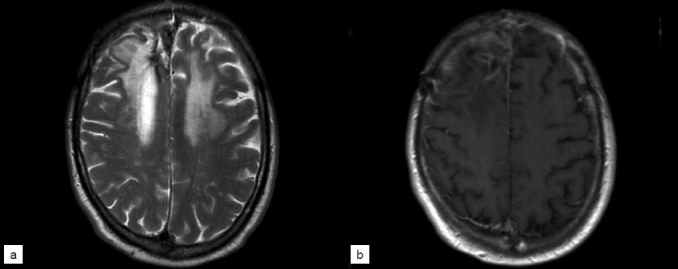
T2 (a) and T1 post-contrast (b) MRI obtained 12 months after craniotomy, showing near-resolution of contrast enhancement with continued evolution of the T2 signal abnormality in the bilateral frontal lobes

Since his craniotomy and diagnosis, the patient has been followed for 18 months. Follow-up MRI studies have demonstrated continued evolution of this process with initial extension of edema to involve the bilateral frontal lobes [Figure 3]. This correlated with increased symptoms of poor balance and cognitive difficulties and was managed with oral steroids. Alternative treatment strategies including anticoagulation were considered but not employed due to contraindications related to the patient’s systemic illness. At 1 year follow-up, imaging studies revealed stabilization of edema and decreased enhancement [Figure 4]. The patient’s clinical status has continued to improve and he remains functionally independent.

## DISCUSSION

The case presented here offers potential insights into the pathogenesis of delayed radiation necrosis. Previously described risk factors for the development of radiation necrosis relate to total radiation dose, dose per fraction, treatment duration and irradiated volume.[10–12] There is also evidence that concomitant or subsequent chemotherapy may be an additional risk factor.[2] This effect may be related to treatment-related immunosuppression and subsequent impaired cellular response to damaged tissue. Risk factors for patients treated for extracranial malignancies, however, are poorly defined. This is due in part to the rarity of this complication in this group. Additionally, rates of reoperation or autopsy are low for this group, which may lead to underdiagnosis of this syndrome.[13]

Cerebral radiation necrosis typically occurs at or near the site of the primary lesion, though contralateral lesions have rarely been reported, as in the case presented here.[14] In such cases, careful analysis of the radiation isodose distribution may demonstrate that the dose to the contralateral hemisphere was higher than expected. In cases such as this one, where the isodose distribution is no longer available, treating physicians must consider that an error in planning or other unknown factors influencing the treatment design may have led to unusually high dosing of the contralateral hemisphere.

The pathogenesis of delayed radiation necrosis is poorly understood. One popular theory postulates that irradiation damages endothelial cells in the brain, leading to breakdown of the blood brain barrier, allowing inflammatory cells to cross into the extravascular space. Their subsequent secretion of cytokines drives further immune responses, leading to a cycle of uncontrolled inflammation.[15] However, newly published data indicate that radiation necrosis rates are higher in patients undergoing cytotoxic chemotherapy.[2] As in the case here, these patients are often immunosuppressed, and this finding indicates that the immune mechanisms involved in the process are more complicated than previously believed.

Symptoms of radiation necrosis are related to mass effect on surrounding structures. Both the clinical presentation and imaging findings can mimic that of a progressive intracranial neoplasm. Differentiating between radiation necrosis and recurrent neoplasm is a frequent challenge for neurosurgeons and neurooncologists. MRI findings are relatively nonspecific. The recent development of two imaging modalities – multivoxel MR spectroscopy and (18)F-FDG-PET – have increased our ability to differentiate between these processes, but the gold standard for diagnosis remains analysis of a pathologic specimen.[16] There are no published studies analyzing the efficacy of these imaging modalities in the diagnosis of cerebral radiation necrosis which occurs in a delayed fashion.[15]

Once accurately diagnosed, the treatment of cerebral radiation necrosis may involve surgical or medical management. Patients with severe symptoms from local mass effect can benefit from debulking of the necrotic lesion.[17] There are also medical strategies which may be employed alone or as an adjunct to surgical treatment. These include systemic steroids, anticoagulation, vitamin E, and hyperbaric oxygen. With or without treatment, the lesion often progresses over time with evolution of imaging findings and clinical symptoms before the process eventually resolves. This natural history makes an accurate diagnosis critical, as it can mimic the progression of an intracranial neoplasm.

While there are no previously reported cases of cerebral radiation necrosis following radiation treatment for a plasmacytoma, previous cases may have been encountered. There are reports in the literature of patients presenting with intracranial mass lesions, years after radiation treatment for extramedullary plasmacytoma of the head and neck (EMPHN) which were presumptively diagnosed as malignant gliomas based on imaging alone.[18] However, the recent review of imaging findings in cerebral radiation necrosis performed by Alexiou *et al*, reminds us that this diagnosis cannot be excluded by imaging alone.[16] Pathologic examination remains the gold standard for differentiating between a radiation-induced malignancy, which may be poorly responsive to therapy, and radiation necrosis, which is a potentially treatable condition. It is possible that symptomatic radiation necrosis has been under-recognized and undertreated in this patient population.

## CONCLUSION

We present the first reported case of cerebral radiation necrosis following radiation treatment for a plasmacytoma. The circumstances are unusual as the complication arose in a delayed fashion and contralateral to the radiation port. This case highlights the fact that cerebral radiation necrosis is a potential complication of radiation therapy for skull lesions and should be considered in the differential diagnosis of any patient with this history and a new intrinsic brain mass, regardless of timing or location. Recognition of this clinical entity by the neurosurgeon is essential, as this potentially treatable condition is best managed with close observation, medical therapy, and surgical intervention for control of mass effect as indicated.

## References

[1] Alexiou GA, Tsiouris S, Kyritsis AP, Voulgaris S, Argyropoulou MI, Fotopoulos AD (2009). Glioma recurrence versus radiation necrosis: Accuracy of current imaging modalities. J Neurooncol.

[2] Babu R, Huang PP, Epstein F, Budzilovich GN (2003). Late radiation necrosis of the brain: A case report. J Neurooncol.

[3] Brandsma D, Stalpers L, Taal W, Sminia P, van den Bent MJ (2008). Clinical features, mechanisms, and management of pseudoprogression in malignant gliomas. Lancet Oncol.

[4] Buge A, Escourelle R, Rancurel G, Gray F, Pertuiset BF (1979). Delayed radionecrosis of the cerebral hemispheres following betatron electron beam irradiation for scalp cancer: Pathological and clinical findings in one case. Sem Hop.

[5] Creach KM, Foote RL, Neben-Wittich MA, Kyle RA (2009). Radiotherapy for extramedullary plasmacytoma of the head and neck. Int J Radiat Oncol Biol Phys.

[6] Glass JP, Hwang TL, Leavens ME, Libshitz HI (1984). Cerebral radiation necrosis following treatment of extracranial malignancies. Cancer.

[7] Kerob D, Kolb F, Margulis A, Mamelle G, Spatz A, Ibrahim M (2002). Delayed cerebral radiation necrosis following radiation therapy of cutaneous squamous cell carcinomas of the head [French]. Ann Dermatol Venereol.

[8] Kumar AJ, Leeds NE, Fuller GN, Van Tassel P, Maor MH, Sawaya RE (2000). Malignant gliomas: MR imaging spectrum of radiation therapy- and chemotherapy- induced necrosis of the brain after treatment. Radiology.

[9] Lee AW, Kwong DL, Leung SF, Tung SY, Sze WM, Sham JS (2002). Factors affecting risk of symptomatic temporal lobe necrosis: Significance of fractional dose and treatment time. Int J Radiat Oncol Biol Phys.

[10] Lee AW, Ng WT, Hung WM, Choi CW, Tung R, Ling YH (2009). Major late toxicities after conformal radiotherapy for nasopharyngeal carcinoma - patient- and treatment- related risk factors. Int J Radiat Oncol Biol Phys.

[11] Lee JK, Chelvarajah R, King A, David KM (2004). Rare presentations of delayed radiation injury: A lobar hematoma and a cystic space-occupying lesion appearing more than 15 years after cranial radiotherapy: Report of two cases. Neurosurgery.

[12] Marks JE, Baglan RJ, Prassad SC, Blank WF (1981). Cerebral radiation necrosis: Incidence and risk in relation to dose, time, fractionation and volume. Int J Radiat Oncol Biol Phys.

[13] Matsumura H, Ross ER (1979). Delayed cerebral radiation necrosis following treatment of carcinoma of the scalp: Clinicopathologic and ultrastructural study. Surg Neurol.

[14] Mikhael MA, Kagan A (1980). Dosimetric considerations in the diagnosis of radiation necrosis of the brain. Radiation damage to the nervous system. A delayed therapeutic hazard.

[15] Ruben JD, Dally M, Bailey M, Smith R, McLean CA, Fedele P (2006). Cerebral radiation necrosis: Incidence, outcomes, and risk factors with emphasis on radiation parameters and chemotherapy. Int J Radiat Oncol Biol Phys.

[16] Sheline GE, Wara WM, Smith V (1980). Therapeutic irradiation and brain injury. Int J Radiat Oncol Biol Phys.

[17] Shewmon A, Masdeu JC (1980). Delayed radiation necrosis of the brain contralateral to original tumor. Arch Neurol.

[18] Yoshii Y (2008). Pathological review of late cerebral radiation necrosis. Brain Tumor Pathol.

